# The New Craig House Asylum, Edinburgh

**Published:** 1894-11-03

**Authors:** 


### WITHIN THE ASYLUMS.

### THE NEW CRAIG HOUSE ASYLUM, EDINBURGH.

On Saturday, the 27th ult., the New Craig House Asylum, in connection with the Royal Edinburgh Asylum for the Insane, was formally opened by the Duke of Buccleuch, governor of the institution. Our views upon this new departure in asylum construction and treatment were fully set forth in The Hospital for October 22nd, 1892, page 62, to which we must refer our readers. We hope to have an early opportunity of inspecting the new institution, but on the present occasion we think it desirable to let Dr. Clouston explain the object and aims of the governors as embodied in this new departure, which promises to effect a revolution in the treatment of the insane. At the opening ceremony, which was a complete success, Dr. Clouston spoke in substance as follows:—

#### Dr. Clouston's Address.

The very first consideration, he said, which occurred to him was this, that it was a disease they had to treat, and therefore the medical aspect was the most important, if not almost the all-important aspect of the building. Everything that had taken place in recent medicine in regard to their subject had shown this, that mental disease was connected with certain definite changes in the brain of the subject who suffered from it. The more they investigated the more definite they became, and now every large institution considered this branch of its work so important that they retained a pathologist, who did nothing else but work at the scientific or medical aspect of this obscure disease, and endeavoured to throw more and more light upon it. The result was that year by year the light of science was thrown with more vividness on their subject, and that they were coming to know something more of it —which meant that it was more amenable, and that they should by-and-by be able to treat it with greater effectiveness than they were able to do at present. In regard to the medical aspect of such a building as the Craig House, he might mention, for instance, that they had now seven medical officers instead of three or four as formerly. He saw the physician of the Berlin Asylum the other day, and he found in comparing it with that institution that they had scarcely enough yet, and that to put it in line with that first-class asylum they should have eight doctors instead of seven. This illustrated better than anything he could say the improved medical surroundings of the patients.

#### The Treatment of Insanity.

Directly the medical treatment of the patient had particularly improved; and there had been within a few years several extraordinary triumphs in the cure of mental disease through direct medical treatment. The recent use of thyroid juice had been little short of miraculous in curing many patients suffering from mental diseases through medical treatment alone. In most diseases something more was needed than the mere administering of physic. There was what might be called the "conditioning" of the patients—both as to body and mind. It was so with mental disease. It was a curious fact that many of their old popular adages were found to be actually of scientific value. It was a long time ago since it was stated that cleanliness was next to godliness; and his friend, Professor Chiene, would bear him out in saying that cleanliness was the foundation of the antiseptic system of surgery and a great factor in preventive medicine. As a matter of science they found that sunlight and brightness had an actual physical effect on the brain of man and woman just as much as an electric magnet had on a needle near it. It stimulated the circulation of the blood to the brain, and produced a more healthful feeling than in cases without light. They had, therefore, in this building endeavoured to the full to take full advantage of this means of cure.

#### The Buildings and their Objects.

Along with light they wanted colour, they wanted a cheerful aspect, they wanted beauty. All these things told in the most direct way on the human brain. He thought he might appeal to them if they had not at all events beauty and colour and cheerfulness in the present hall. They found also that it was said that "A merry heart doeth good like a medicine." They required to provide for the social wants of their patients, and that would be done by the concerts and other entertainments they hoped to have in this hall. They all knew that some people were naturally of a cheerful disposition; but every one had not got that, and those who had not got it, and became patients here, required by every effort of those about them, and by every means they could employ in the building, to have that kind of feeling produced. In the olden time Marcus Aurelius said that "Gentleness is invincible," and a greater than Marcus Aurelius had said that "a soft answer turneth away wrath." Those two precepts they had endeavoured to embody in the life of this institution and in the treatment of the patients. They had in this form of disease an irritation, and the application of hard words was simply an extra irritant, so that this adage of the "soft answer" came in as an actual part of medical treatment. Then as to the institution, those who knew old Craig House and did not think that it was charming, and that the views from its elevated site, and the magnificence of the panorama it commanded of the city of Edinburgh and the Firth of Forth, must be cheering and stimulating—well, he had nothing more to say to them.

### The Men and the Building.

Having a good site beautifully wooded, the next thing theyhad to consider was the erection of a building which would embody the most recent scientific requirements. He went and saw several institutions in Europe and in America, and in this country. He saw a great deal to imitate, and many things to avoid. He was delighted to see among them that day from Manchester their friend Mr. Mould, who more than anybody else in England had laboured to ameliorate the condition of the insane. He also saw present Sir Arthur Mitchell, who had done more for the mentally afflicted in Scotland than anybody in this hall could believe, unless they were acquainted with the actual facts of the case. As to their architect, Mr. Sydney Mitchell, he (Dr. Clouston) might say once for all that a more loyal architect to medical principles never existed, and he had had the genius to give them at once a first-rate medical institution and a real work of art, which it was not given everybody to produce. The older institutions of this kind had been planned chiefly with the view to restraint. They had got over that. What they had to do was to suit the building to the mental condition of the patients. It was absolutely out of the question to put all under one roof. They would see by the block plan which had been put into their hands that this was not one building but a series of buildings, each differing from the other in aspect so as to suit them to the varying mental conditions of those who were to occupy them. They had in this building a counteractive to mental disease in stone and lime. Innumerable prejudices, as they knew, still lingered in the popular mind. This building was a popular object lesson in the new treatment of mental disease. They wished to make it known to all men that this thing had changed, and that the change was in the right direction. To provide for the social needs of the patients they had to provide a room in which a large number of people could meet together. Everyone who had lived in a country mansion knew what a charming place the hall was, where everybody met on a common standing ground, and which was the centre of the social life of the mansion. They had in this grand hall given to the mentally afflicted the benefits of the common hall of a Tudor mansion. Opening off it were the drawing-room and billiard-room, and at the other end the dining-rooms; and outside and inside the building they would everywhere find great variety. That was one of the great principles on which they had worked.

### The Hospitals and Nursing.

In their perambulation they would see that they had erected two separate hospitals for those who were physically weak and needed nursing; and that to many was a great part of their treatment. They had gone in here for separate hospitals, and the Commissioners of Lunacy had approved of the idea, which had also been adopted in other institutions in Scotland. They had also Bevan House, one of their villa residences, in which anyone might reside. As to the nursing department, that was one of the most important of all the means of treatment they could possibly have. Hitherto they had had a very good staff of nurses, but lately the Medico-Psychological Association had set up a special system of training for nurses, and he was pleased to say that forty of their staff had passed the examination and held certificates in practical psychological or mental nursing as given by this association.

### An Object Lesson.

In reference to the administration, they had endeavoured to administer the institution on principles of order combined with liberty, and he hoped common-sense, firmness, and kindness Civilisation had brought them many advantages, but he was afraid that their modern civilisation had brought them also, to a certain extent, certain forms of brain trouble, and they therefore called upon civilisation to undo the evils that it had brought in its train. If this institution was not required in a few years for the purpose for which it had been built, he believed it could be turned at little cost into a hotel or nobleman's mansion. Some might say that they had overdone things a little. He thought that a little overdoing might be pardoned, for they had atonement to make for the cruelty and the neglect of past ages. When the history of the end of the nineteenth century came to be written and the various social evolutions going on around them came to be described, he hoped this building would be a standing evidence that the people of this period had not neglected the mentally afflicted, that they had done here all that the medical science of the time knew to do; and they fervently trusted that what this building did it would do effectively for the cure of the most painful of all diseases.

